# Midwives’ experiences of care provision in refugee camps under the guidelines of the ORAMMA project: a qualitative study

**DOI:** 10.1017/S1463423626101492

**Published:** 2026-07-24

**Authors:** Eleni Triantafyllou, Maria Papadakaki, Elena Petelos, Antigoni Sarantaki, Anna Deltsidou, Victoria Vivilaki

**Affiliations:** 1 Midwifery, University of West Attica, Athens, Greece; 2 Social Work, Hellenic Mediterranean University, Crete, Greece

**Keywords:** challenges, midwives, refugee camps, resilience

## Abstract

**Aim:**

This study aimed to explore the experiences of midwives providing care in primary care walk-in clinics (PCWCs) within refugee camps, examining the challenges they encounter, and the relevance the ORAMMA framework.

**Background:**

Refugee camps present a complex environment for maternal care provision, due to limited resources, high population mobility, cultural and linguistic diversity, and increased psychosocial vulnerability among women. Midwives often being the primary providers of maternal care, must address both social and clinical needs. The ORAMMA framework was developed to support culturally sensitive and equitable maternity care for migrant and refugee populations. However, limited evidence exists regarding field experiences of ORAMMA-guided midwifery practice.

**Methods:**

A qualitative study design was employed to capture midwives’ in-depth experiences. Data were collected from 22 ORAMMA-trained registered midwives working in refugee camp PCWCs. Data were collected using semi-structured individual interviews (*n* = 15) and two focus groups (*n* = 7). Data were thematically analysed to identify recurring patterns and key themes.

**Findings:**

Midwives reported persistent challenges, including inadequate infrastructure, shortages of essential medical supplies and disrupted care continuity. Language and cultural barriers limited effective communication, trust-building, and clinical decision-making. Despite these constraints, midwives demonstrated adaptability and professional resilience, through collaborative teamwork, creative problem-solving, and culturally sensitive care. ORAMMA training strengthened their confidence and awareness in providing care, although participants emphasised the need for enhanced material resources, interpreter services, and psychosocial support. Overall, findings highlight the value of ORAMMA-guided capacity-building to support midwives’ resilience and commitment to quality care delivery.

## Background

Forced displacement has increased markedly over the past decade due to armed conflict, persecution, and human rights violations. According to the United Nations High Commissioner for Refugees (UNHCR), more than 120 million people worldwide were forcibly displaced by the end of 2024, highlighting the sustained and global nature of the migration and refugee crisis (UNHCR, 2025). Within Europe, Greece has remained a frontline country since 2015, facing ongoing challenges in providing adequate healthcare services to refugee and asylum-seeking populations, particularly in reception and camp settings (Gunst *et al*., 2019).

It is noted that refugees and asylum seekers commonly experience significant barriers to accessing healthcare, including administrative obstacles, fragmented services, and communication difficulties (Morris *et al*., 2009; Robertshaw *et al*., 2017; Sherif *et al*., 2022). These challenges are particularly pronounced in maternity care, where effective communication, continuity, and trust are critical to clinical safety and quality. Evidence from Greece and other host high-income countries (HICs) indicates that language barriers, limited availability of interpreters, and cultural distance can compromise access to timely and appropriate perinatal care (Betancourt *et al.,* 2003; Alizadeh and Chavan, 2016; Al Shamsi *et al*., 2020; Scott and Wallis, 2021).

Many researchers highlight how refugee women are at increased risk of adverse maternal and perinatal outcomes, including stillbirth, preterm birth, and selected pregnancy-related complications, compared with host populations in HICs (Carolan, 2010; Harakow *et al*., 2021). Delayed initiation of antenatal care and reduced engagement with maternity services have been consistently associated with lack of familiarity regarding host-country healthcare systems, cultural differences, and communication barriers. In camp-based settings, these risks are further intensified by resource constraints and disrupted care pathways (Kentoffio *et al*., 2016). In the Greek camp context, care was commonly provided through primary care walk-in clinics (PCWCs ) located within refugee camps, where midwives offered assessment without appointment requirements (Gunst *et al.*, 2019; Scott and Wallis, 2021).

Midwives play a central role in addressing the maternity care needs of refugee women in these contexts. They ensure essential antenatal care, monitoring pregnant women’s health to manage complications and promote maternal and infant well-being through screenings, health education and family planning, and, critically, through timely vaccinations (McFadden *et al.*, 2021). Their role extends to postnatal support, where they address physical and emotional health issues, promote breastfeeding, and guide maternal recovery (Tanabe *et al.*, 2019). Furthermore, they have a role to play in reproductive health education, family planning, nutrition, and hygiene awareness, empowering women and girls with knowledge to improve their health outcomes (McGinn *et al.*, 2017). Additionally, they facilitate referrals for complex cases requiring higher-level care and work alongside other healthcare providers and organisations to advocate for improved maternal and infant health services in refugee settings (Kebede *et al*., 2020; Rogers *et al*., 2020; Dey *et al*., 2024). Beyond physical care, midwives provide emotional and psychological support, addressing trauma, anxiety, and other mental health concerns prevalent in crisis situations (van der Boor and White, 2020).

Despite their crucial role, they face numerous challenges that hinder their efficiency and effectiveness in delivering quality care in PCWCs in refugee camps. For instance, language barriers remain a significant obstacle (Robertshaw *et al*., 2017; Barrio-Ruiz *et al*., 2023), preventing clear communication between midwives and refugee women and leading to misunderstandings about healthcare services and maternal health status (Dowling *et al*., 2020). Poor intercultural communication further exacerbates this issue, limiting satisfaction among medical professionals and patients and reducing healthcare quality (Al Shamsi *et al*., 2020). Additional challenges include poor facilities and inadequate resources within the PCWCs, a lack of specialised training for midwives in emergency and crisis settings, and insufficient collaboration between healthcare professionals and policymakers (Triantafyllou and Vivilaki, 2023). These barriers restrict access to timely and appropriate maternal healthcare, leading to adverse maternal and neonatal outcomes.

To address these challenges, midwives require targeted and continuous training adapted to resource-limited and humanitarian settings, with emphasis on emergency obstetric care, trauma-informed practice (Eckstein and Culhane-Pera, 2020), and cultural competence (Seng *et al*., 2008; McGinn *et al*., 2017; Iliadou *et a*l., 2019; van der Boor and White, 2020). Strengthening collaboration with community actors and interpreters can enhance trust and accessibility of maternal services, while partnerships with non-governmental organisations and public authorities are essential to secure resources and workforce support (Tanabe *et al*., 2019; Kebede *et al*., 2020). At a systemic level, advocacy and routine data collection on maternal outcomes are needed to inform policy, guide resource allocation, and support sustainable investment in midwifery-led care for refugee populations (McFadden *et al*., 2021).

The ORAMMA (Operational Refugee and Migrant Maternal Approach) project was implemented in Europe aiming to improve maternal care services, particularly for vulnerable refugees and migrant women. Midwives working in PCWCs under ORAMMA guidelines played a crucial role in providing comprehensive, culturally adapted care, as they were trained to understand and respect cultural differences, emphasising the use of interpreters and cultural mediators to enhance communication and better address women’s needs (ORAMMA Project Consortium, 2018). ORAMMA midwives thereafter educated and supported pregnant women and new mothers about healthcare, childbirth, and newborn care while also preventing and managing pregnancy complications (Triantafyllou *et al.*, 2024). Their training covered the perinatal care needs of refugee women, including psychological and emotional support in collaboration with mental health professionals (Huschke and Coe, 2019). The ORAMMA guidelines enabled midwives to detect and address health needs early through structured protocols, focusing on nutrition, infection prevention, and sexually transmitted disease management (Delara, 2016). Midwives provided individualised care, advising mothers on breastfeeding, infant care, and post-pregnancy management, while also fostering women’s empowerment through support networks and social assistance programmes (Van den Muijsenbergh and Van Weel-Baumgarten, 2015). The ORAMMA approach underscored the holistic role of midwives – not only as clinical caregivers but also as advocates and social support providers, critical for refugee and migrant women facing increased vulnerability during pregnancy and childbirth.

Overall, midwives are indispensable in refugee settings, bridging the gap between limited healthcare resources and the urgent maternal health needs of displaced women. Their work ensures that vulnerable populations receive essential care, contributing to improved maternal and child health outcomes despite challenging conditions. Against this background, this study sought to examine the lived experiences of midwives working in PCWCs in refugee camps in Greece in order to better understand the existing challenges and the ways in which ORAMMA training informs their clinical practice.

To address the study’s aim and to gain a deeper understanding of midwives’ experiences in refugee-camp-based primary care settings, a set of exploratory research questions was developed. These questions focused on the challenges encountered in everyday clinical practice, the strategies used to navigate communication and organisational barriers, and the perceived contribution of ORAMMA training to the provision of culturally sensitive maternity care.

1. How do midwives describe their experiences of providing perinatal care within refugee camp-based PCWCs?
2. What challenges do midwives encounter when caring for refugee and migrant women in these settings?
3. How do midwives manage communication, cultural, and organisational barriers in everyday practice?
4. How do midwives perceive the contribution of ORAMMA training to their clinical practice and care provision?

## Methodology

### Study design

The study employed a qualitative methodology with semi-structured interviews and focus groups, as it aims to explore participants’ experiences, perspectives, and beliefs in depth. Semi-structured interviews provide a flexible framework where the researcher uses predefined questions but allows room for follow-up questions or exploration of unanticipated topics based on the participant’s responses (Yoost and Crawford, 2022). This qualitative study involved semi-structured one-to-one interviews (Dowling *et al.*, 2020) of 15 midwives working in PCWCs established in refugee camps located in different regions and in conducting two focus groups consisting of four and three midwives respectively (seven midwives in total).

### Participants and recruitment

Participants were registered midwives eligible to practise in Greece, with professional experience in providing care to migrant and refugee populations in PCWCs located within refugee camps, including governmental services and non-governmental organisations (NGOs). Recruitment was conducted at a national level through email distribution lists and professional networks, including midwifery regulatory and public health associations in Greece. Additional recruitment support was provided by the Hellenic Center for Disease Control and Prevention (HCDCP/KELPNO) the HCDC was Greece’s public health organisation until 2019, fully transitioning to its current form as the National Public Health Organisation (NPHO/EODY) through the physician responsible for the relevant services. Participation was voluntary. To enhance the diversity and richness of the data, purposive sampling was applied, duly considering years of professional experience, type of service setting, and the volume of migrant women cared for in daily practice.

### Training and professional background

All participating midwives had completed training through the ORAMMA (Operational Refugee and Migrant Maternal Approach) project, which aimed to strengthen competencies in culturally sensitive perinatal care for migrant and refugee populations (Petelos *et al*., 2019). This training equipped participants with knowledge and skills related to communication, cultural mediation, and the management of perinatal care in resource-limited and humanitarian contexts. Participants were primarily engaged in healthcare services supporting migrant populations.

### Data collection

Data were collected from a total of 22 midwives. Fifteen participants took part in individual semi-structured interviews, while seven participated in two focus groups comprising four and three midwives, respectively.

Individual interviews and focus groups were conducted face-to-face by members of the research team experienced in qualitative interviewing. Individual interviews lasted approximately 20 minutes on average, while focus groups lasted approximately 60 minutes. Participants received information about the ORAMMA project prior to the informed consent process and to providing agreement to record. A brief introduction to the study was also provided at the beginning of each interview and focus group. Data collection was facilitated by an experienced qualitative researcher using a prespecified interview guide while allowing opportunities for participants to provide additional comments and insights. All interviews and focus groups were audio-recorded, transcribed by a second researcher, and reviewed by the facilitator to ensure accuracy and completeness. The combination of these methods enhanced the depth and breadth of the data and supported a comprehensive understanding of midwives’ experiences in refugee maternal healthcare.

A semi-structured interview guide and focus group topic guide were used to ensure consistency while allowing participants to elaborate on issues of importance. The guides are provided as Supplementary File 1.

### Ethical approval

Ethical approval for the study was obtained in accordance with the legislative framework in Greece (Reference number: 5154/12-03-2018, General Hospital (G.H.) ‘Elena Venizelou-Alexandra’). All participants were informed about the aims of the study, and informed consent was obtained prior to data collection.

### Data analysis

Data were analysed using thematic analysis, following the approach described by Braun and Clarke (2006). This process involved familiarisation with the data, systematic coding, and the identification and refinement of themes related to participants’ experiences, challenges, and coping strategies.

## Results

The thematic analysis identified a set of interrelated themes reflecting the complexity of midwives’ practice in primary care walk-in clinics within refugee camp settings. These themes encompassed structural and organisational constraints, communication and relational challenges, disruptions in continuity of perinatal care, and the emotional and ethical dimensions of care delivery. In addition, participants described experiences of professional resilience and reflected on the perceived value and limitations of ORAMMA training in supporting practice in resource-constrained environments. The themes are presented below:

### Resource scarcity and inadequate working conditions

Midwives consistently reported severe shortages in medical supplies, equipment, and appropriate clinical infrastructure. Limited access to essential medications, lack of privacy in consultation spaces, and poorly equipped facilities were described as routine rather than exceptional conditions. These constraints were perceived as directly affecting both the safety and quality of perinatal care, forcing midwives to improvise and prioritise urgent needs over comprehensive assessment and follow-up. The physical environment itself was often described as undermining women’s sense of dignity and comfort during care encounters.

*‘The lack of equipment, supplies and inadequate amenities’. (Midwife 10, Interview)*

1. **Communication barriers and cultural distance**

Language barriers emerged as one of the most critical challenges in providing effective maternal care. Participants highlighted the limited availability of trained interpreters and cultural mediators, which frequently hindered accurate history taking, informed consent, and health education. Communication difficulties were further compounded by cultural differences in perceptions of pregnancy, childbirth, and healthcare utilisation. Several midwives emphasised that the absence of female interpreters posed additional challenges when discussing sensitive reproductive or gynaecological issues, increasing the risk of missing clinically relevant information.

*‘The lack of interpreters was also a problem and especially the lack of female interpreters – these women are not comfortable to discuss female issues in front of men, which can result in missing important information’. (Midwife 7, Interview)*

1. **Building trust in contexts of trauma and insecurity**

Establishing trust with refugee women was described as a complex and time-consuming process. Midwives perceived that many women had experienced significant trauma and uncertainty, which influenced their interactions with healthcare professionals. Distrust, fear, and occasional expressions of distress or aggression were interpreted not as resistance to care, but as manifestations of prior traumatic experiences and ongoing insecurity. Trust-building was therefore viewed as a prerequisite for effective care, requiring patience, empathy, and continuity – elements often constrained by the camp setting.

*‘They were also suspicious because of [the] racism they probably face. It was hard for them to trust me’. (Midwife 14, Interview)*

1. **Disrupted continuity of perinatal care**

Participants described significant challenges in ensuring continuity of care across the antenatal, intrapartum, and postnatal periods. Limited follow-up, difficulties in referral pathways, and reluctance or inability of women to attend hospital appointments were commonly reported. Weak links between camp-based services and hospital care, particularly in the absence of interpreters, further disrupted care trajectories. As a result, midwives often felt unable to monitor pregnancies longitudinally or intervene early when complications arose.

*‘Another challenge was to ensure follow-ups during the perinatal period. It was hard to keep continuous contact with these women, as many faced difficulties attending hospital appointments. Cultural attitudes toward perinatal care, distance between the hospital and the camps, childcare issues, family visits, and relocation often prevented them from returning for follow-up, which sometimes resulted in inadequate antenatal and postnatal care’. (Focus Group 1)*

1. **Emotional burden, moral distress, and professional resilience**

Working in PCWCs in refugee camps was associated with a substantial emotional burden. Midwives described exposure to distressing narratives, high workloads, and the frustration of providing care under suboptimal conditions. Feelings of moral distress were particularly evident when participants felt unable to deliver care aligned with their professional standards and moral codes. At the same time, midwives demonstrated resilience, drawing strength from teamwork, peer support, and the perceived meaningfulness of their role. Informal collaboration with colleagues and multidisciplinary teams was frequently cited as a key protective factor allowing them to safeguard their professional resilience.

*‘It is extremely hard to provide quality perinatal care in such settings mostly because they are usually understaffed but also as far as the facilities are concerned, they are poorly constructed and offer low capacity in relation to the required’. (Midwife 2, Interview)*

1. **Perceived quality of care and professional standards**

While some midwives felt able to provide compassionate and supportive care despite constraints, many expressed concerns that the quality of care delivered did not meet their professional expectations. Participants distinguished between offering humanitarian support – such as empathy, reassurance, and emotional presence – and delivering comprehensive, high-quality clinical care. Limitations in equipment, communication, and infrastructure were perceived as major barriers to achieving the latter, reinforcing feelings of professional inadequacy despite strong personal commitment.

*‘I feel that I can provide humanitarian help (empathy, kindness) but sometimes due to the lack of equipment and due to the lack of direct communication (language barrier) I might not be able to offer the quality of care I want to’. (Midwife 4, Interview)*

1. **Perceived value of ORAMMA training**

The majority of participants described the ORAMMA training as beneficial, particularly in enhancing communication skills, cultural awareness, and confidence in working with refugee women. The training was perceived as especially valuable for midwives with limited prior experience in migrant health settings. However, some participants indicated that more experienced midwives would benefit from advanced, practice-oriented training addressing the complexity and unpredictability of camp-based care. The communication module was consistently highlighted as a core strength of the training programme.

Overall, data analysis revealed several key themes, including inadequate resources, cultural challenges, emotional and physical stress, and resilience. Midwives reported difficulties in accessing medical supplies and providing culturally sensitive care. Despite these challenges, they demonstrated remarkable resilience and adaptability. Key messages that emerged were good multidisciplinary teamwork with obstetricians and midwives, as in refugee and migrant maternal healthcare effective collaboration between obstetricians and midwives is crucial. The pivotal role of midwives also emerged as midwives play a central role in maternal healthcare, particularly within refugee populations. Key responsibilities include health advocacy, supporting clinically and emotionally complex cases, cultural sensitivity, language and communication support. As for the challenges faced in refugee maternal care, lack of specific guidelines for refugee health, language difficulties and cultural barriers, midwifery workforce shortages, limited resources and supplies and poor-quality facilities were reported.

*‘I felt that the training was enough for healthcare professionals who had never worked with refugee populations before, but for those of us with more experience, a more detailed training would have been helpful’. (Focus Group 2)*

*‘Yes, it was my first time working with refugee women and the ORAMMA training helped me a lot. The communication module was a lifesaver’. (Midwife 15, Interview)*

### Trustworthiness and rigour

Trustworthiness was enhanced through the use of multiple qualitative data sources, including individual interviews and focus groups with midwives working in primary care walk-in clinics within refugee camps. This methodological triangulation allowed for convergence and depth of understanding across different forms of professional experience. The analytic process followed an iterative approach, involving repeated reading of transcripts, systematic coding, and collaborative theme development. Reflexive discussions within the research team supported consistency and transparency in interpretation. Thick, contextualised descriptions and illustrative excerpts were used to support the credibility and transferability of the findings.

## Discussion

This study provides insight into the complex realities faced by midwives delivering perinatal care in PCWCs in refugee camp settings. The findings highlight how structural deficiencies, communication barriers, and fragmented care pathways intersect to shape everyday clinical practice. While these challenges are well documented in the broader literature on refugee health, the present analysis underscores how they are experienced and navigated at the frontline of maternal care, where professional responsibility, ethical commitment, and systemic constraints frequently collide (Robertshaw *et al*., 2017; Rogers *et al*., 2020).

Consistently with previous research, inadequate infrastructure and shortages of essential supplies at PCWCs emerged as central barriers to the provision of safe and effective maternal care. Resource scarcity was not perceived as an occasional disruption but as a systemic issue, i.e., persistent conditions requiring continuous adaptation in terms of practice and care provision (Triantafyllou and Vivilaki, 2023; Triantafyllou *et al.,* 2023; Dey *et al*., 2024). Such limitations have been associated with increased clinical risk, compromised privacy, and reduced capacity for comprehensive assessment and follow-up. For midwives, these constraints contributed to moral distress, particularly when the care provided fell short of professional standards despite their best efforts.

Communication barriers were identified as a critical determinant of care quality and patient safety (Robertshaw *et al*., 2017; Barrio-Ruiz *et al*., 2023). The limited availability of trained interpreters and cultural mediators constrained accurate clinical assessment, informed consent, and health education. Importantly, communication was not framed as a ‘soft’ or ancillary issue but as a core clinical requirement. The absence of female interpreters, in particular, was perceived as a significant obstacle to addressing sensitive reproductive and gynaecological concerns, echoing findings that link gender-concordant communication to improved disclosure and engagement in maternal care.

Trust emerged as a central yet fragile component of effective perinatal care in refugee settings. Participants’ accounts suggest that women’s reluctance, emotional volatility, or disengagement from care should be understood within the context of trauma, displacement, and prolonged uncertainty rather than as non-compliance. Establishing trust required time, continuity, and relational consistency – elements that are often difficult to sustain in camp-based services. These findings align with trauma-informed care frameworks, which emphasise the creation of physical and emotional safety, collaborative and trusting relationships, and service-user empowerment as essential foundations for engagement (CSAT, 2014; GOV.UK, 2022; Edelman, 2023).

Disrupted continuity of care was a recurring concern, particularly across antenatal, intrapartum, and postnatal transitions. Weak referral pathways, limited coordination with hospital services, and language barriers contributed to fragmented care trajectories. Such disruptions undermine early detection of complications and continuity-based interventions known to improve maternal and neonatal outcomes. The findings point to systemic gaps rather than individual professional shortcomings, reinforcing the need for integrated service models that bridge camp-based and secondary care.

The emotional demands of working in refugee camps were evident across participants’ narratives. Exposure to traumatic stories, high workloads, and constrained working conditions contributed to emotional exhaustion and moral distress (Hunter and Warren, 2014). Nevertheless, midwives demonstrated resilience through professional solidarity, peer support, and a strong sense of purpose. Teamwork and informal support networks functioned as protective factors, enabling midwives to sustain engagement despite ongoing stressors (Paloma *et al.,* 2020). These findings reflect broader evidence linking meaning in work and collegial support to resilience in high-burden healthcare settings.

### The role and limits of ORAMMA training

The ORAMMA training programme was widely perceived as a valuable resource, particularly in strengthening communication skills, cultural awareness, and confidence in working with refugee women. Its emphasis on culturally sensitive care and structured guidance supported midwives in navigating complex clinical and relational challenges. However, the findings also suggest that training alone is insufficient to offset systemic deficiencies and to compensate for structural barriers (Lionis *et al.,* 2018). More advanced, context-specific education addressing trauma-informed care, complex case management, and ethical decision-making in resource-limited environments may be required, particularly for experienced practitioners. Further research in terms of how the experiences of care recipients shape their health-seeking behaviours in the host HIC, within and beyond these settings, is also warranted.

The excerpts demonstrate that ORAMMA training contributed to increased cultural awareness and communication confidence, particularly among healthcare professionals with limited experience of caring for refugees. Participants with more extensive prior experience, however, expressed a preference for more advanced and detailed training. Furthermore, participants highlighted that education alone cannot address systemic barriers and stressed the importance of adequate resources, interpreter provision, and integrated referral mechanisms.

### Implications for practice and policy

Taken together, the findings highlight the need for multi-level interventions to support midwives working in refugee settings. Investments in infrastructure, interpreter services, and integrated care pathways are essential to improve care quality and safety. Equally important is the provision of ongoing professional development and emotional support for midwives exposed to sustained occupational stress. At a policy level, the absence of clear national guidelines tailored to refugee maternal health emerged as a critical gap, underscoring the importance of structured frameworks that translate humanitarian principles into operational practice.

## Conclusion

This study highlights that midwives working in refugee camp settings face persistent structural, organisational, and relational challenges that significantly affect the delivery of perinatal care. Resource scarcity, communication barriers, disrupted continuity of care, and emotional strain shape everyday practice, often limiting midwives’ ability to provide care aligned with professional standards. Despite these constraints, participants demonstrated strong resilience, professional commitment, and adaptability, frequently compensating for systemic gaps through relational, woman-centred approaches. These findings align with existing evidence indicating that migrant and refugee women in Europe remain at increased risk of adverse maternal and perinatal outcomes, underscoring the critical role of skilled and supported maternity care providers in these contexts (Fair *et al*., 2020).

Strengthening culturally competent, trauma-informed maternity care requires sustained investment in material resources, interpreter services, and targeted professional development for healthcare staff. International guidance emphasises that improving health outcomes for migrant and refugee women depends on enhancing healthcare providers’ confidence, empowering them through context-relevant capacity-building regarding communication skills, and cultural awareness, alongside supportive organisational and policy frameworks (World Health Organization, 2018). The findings of this study further suggest that structured training initiatives, such as those offered through the ORAMMA project, can enhance midwives’ preparedness and confidence, particularly in relation to cross-cultural communication and care for vulnerable populations (Petelos *et al.*, 2019). However, training alone is insufficient to address the complex array of barriers and obstacles without parallel system-level support addressing workforce capacity, infrastructure, and continuity of care.

### Implications and future research

Future research should extend beyond single professional groups to include larger and more diverse samples of healthcare professionals across different healthcare settings in order to inform integrated models of refugee healthcare delivery (Kelley *et al*., 2018; Sheikh and MacIntyre, 2020). Mixed-methods and participatory approaches, incorporating refugees’ own perspectives, could further illuminate the complex interaction between health needs, social determinants, and healthcare access (Riley *et al*., 2017; Woodgate *et al*., 2017). In parallel, evaluative research is needed to assess the effectiveness of evidence-based interventions focusing on cultural competence, trauma-informed care, communication support, and peer-mediated models of care (Betancourt *et al*., 2016; Ismail *et al*., 2018). Such work may support the development of sustainable, data-informed strategies to improve maternal and child health outcomes for refugee populations within integrated primary healthcare systems (Epstein and Street, 2011).

## Supporting information

10.1017/S1463423626101492.sm001 Triantafyllou et al. supplementary material Triantafyllou et al. supplementary material

## Data Availability

The data supporting the findings of this study consist of anonymised interview transcripts generated during the ORAMMA project. Due to the qualitative nature of the data and the need to protect participant confidentiality, the data are not publicly available. Anonymised transcripts may be made available from the corresponding author upon reasonable request and subject to ethical approval and data protection regulations.

## Supplementary material

The supplementary material for this article can be found at https://doi.org/10.1017/S1463423626101492.
