# Midwives’ experiences of care provision in refugee camps under the guidelines of the ORAMMA project: a qualitative study – ADDENDUM

**DOI:** 10.1017/S1463423626101820

**Published:** 2026-08-06

**Authors:** Eleni Triantafyllou, Maria Papadakaki, Elena Petelos, Antigoni Sarantaki, Anna Deltsidou, Victoria Vivilaki

**Affiliations:** 1 Midwifery, University of West Attica, Athens, Greece; 2 Social Work, Hellenic Mediterranean University, Crete, Greece; 3 HSR, CAPHRI Care and Public Health Research Institute, Faculty of Health, Medicine and Life Sciences, Maastricht University, Maastricht, The Netherlands.; 4 Clinic of Social and Family Medicine, School of Medicine, University of Crete, Heraklion, Crete, Greece.

When this article was originally published, two of Elena Petelos’ affiliations were inadvertently missed:

- HSR, CAPHRI Care and Public Health Research Institute, Faculty of Health, Medicine and Life Sciences, Maastricht University, Maastricht, The Netherlands.
- Clinic of Social and Family Medicine, School of Medicine, University of Crete, Heraklion, Crete, Greece.

The authors apologise for this error. The original article has now been updated.
