# Supplementary material for: Midwives’ experiences of care provision in refugee camps under the guidelines of the ORAMMA project: a qualitative study
Source: Prim Health Care Res Dev. 2026 Jul 24;27:e77. doi: 10.1017/S1463423626101492 (PMC13434055; doi:10.1017/S1463423626101492)
Supplement: Supplementary file 1 [file S1463423626101492sup001.docx]

**Interview guide**

1. What are the challenges of working as a midwife in refugee camps?

2. Do you feel you are able to provide quality perinatal care in such settings?

3. What do you think could be improved in order for you to provide the best possible care for

refugee women?

4. Do you feel there are enough sources to support you when working in such settings? (e.g.

guidelines, legislation)

5. Did you find the ORAMMA training helpful for your role as a midwife working with refugee

women?

**Focus Group Questions**

1. What was your overall experience with the ORAMMA project?

2. What was your experience of the ORAMMA training?

3. Do you have any recommendations for future improvement of the ORAMMA training?
